# 基于金属/共价有机框架分子印迹材料的固相萃取结合色谱/质谱在海岸带新污染物筛查识别中的应用

**DOI:** 10.3724/SP.J.1123.2025.03010

**Published:** 2025-08-08

**Authors:** Jingying HUANG, Jingyi YAN, QI Ji, Lingxin CHEN, Jinhua LI

**Affiliations:** 1.中国科学院烟台海岸带研究所，山东省海岸带环境过程重点实验室，海岸带生态环境监测技术与 装备山东省工程研究中心，山东 烟台 264003; 1. Coastal Zone Ecological Environment Monitoring Technology and Equipment Shandong Engineering Research Center，Shandong Key Laboratory of Coastal Environmental Processes，Yantai Institute of Coastal Zone Research，Chinese Academy of Sciences，Yantai 264003，China; 2.中国科学院大学，北京 100049; 2. University of Chinese Academy of Sciences，Beijing 100049，China

**Keywords:** 海岸带, 新污染物, 筛查识别, 固相萃取, 分子印迹, 金属有机框架, 共价有机框架, 复合材料, coastal zone, new pollutants（NPs）, screening and recognition, solid-phase extraction（SPE）, molecular imprinting, metal-organic frameworks（MOFs）, covalent-organic frameworks（COFs）, composite materials

## Abstract

海岸带作为海陆交互作用的关键区域，承载着复杂的生态系统与高强度的人类活动。随着工业化和城市化的快速发展，持久性有机污染物（POPs）、内分泌干扰物（EDCs）、抗生素、微塑料等新污染物不断涌入海岸带。这些污染物具有浓度低、种类多、化学性质复杂等特点，对海岸带生物多样性及人类健康构成严重威胁。对新污染物进行筛查识别和精准检测是研究其环境行为、健康危害及消减控制等的首要前提，相关研究将为后者提供强有力的理论和技术支撑。然而，海岸带样品基质复杂（如水体、沉积物、土壤、生物样品、大气等多类型介质；高盐度、共存的多物种干扰等），通常采用的高灵敏的色谱或色谱-质谱检测技术仍然需要结合高效的样品前处理。基于功能材料的固相萃取（SPE）已成为重要的样品前处理手段，例如具有构效预定性的分子印迹聚合物（MIPs）凭借其“锁钥”特异性识别机制在SPE中备受青睐。然而，MIPs仍存在模板分子残留/泄漏、吸附容量低、传质速率低及复杂基质适应性欠佳等困扰，制约其实际应用。近年来，高比表面积与可调孔径的金属/共价有机框架（MOFs/COFs）材料的引入为MIPs性能提升提供了新思路，可显著增强MIPs的吸附容量和传质速率及适用性等。因此，MOF/COF-MIPs复合材料日益引起关注，其作为SPE吸附剂在海岸带新污染物筛查识别中占据一席之地。本文综述了MOF/COF-MIPs这两类复合材料结合SPE前处理及色谱/质谱测定用于海岸带新污染物筛查识别的研究新进展，概述了MIPs的制备方法并着重讨论了分子印迹固相萃取（MISPE）中需要考虑的吸附容量、结合动力学传质速率、选择性和抗干扰能力等关键因素。同时，介绍了MOF-MIPs和COF-MIPs复合材料的制备，聚焦MOF/COFs的结构优势如可设计性等及其对MISPE性能的提升作用。本文还梳理了MOF/COF-MIPs-SPE结合色谱或色谱-质谱在海岸带POPs、EDCs、抗生素、微塑料等新污染物测定中的典型应用。最后，提出了两类复合材料在海岸带新污染物检测中面临的挑战，并展望了其制备和应用前景。

## Abstract (en)

The coastal zone， a critical ecotone where marine and terrestrial environments converge， contains complex ecosystems， is subjected to highly intense human activity， and is experiencing escalating threats from anthropogenic pollutants. Rapid industrial and urban development has witnessed the continual entry of new pollutants（NPs）， such as persistent organic pollutants（POPs）， endocrine-disrupting chemicals（EDCs）， antibiotics， and microplastics， into coastal zones. These NPs are present in low concentrations in the environment， are structurally diverse and chemically complex， and pose severe risks to biodiversity and human health. Methods for effectively screening， recognizing， and precisely determining NPs are urgently required to understand their environmental fate and toxicological impact， and to formulate abatement-control strategies. However， the intrinsic complexities of coastal matrices， as characterized by heterogeneous media（such as water， sediments， soil， biological components， and the atmosphere）， high salinity， and interference from a variety of species， demands advanced sample-pretreatment methodologies coupled with highly sensitive chromatography or chromatography-mass spectrometry（MS） techniques. Functional-material-based solid-phase extraction（SPE） has become an important sample-pretreatment method. Molecularly imprinted polymers（MIPs） that operate through “lock-and-key” molecular recognition， have become very popular in the SPE space. Nevertheless， MIPs suffer from issues such as template-molecule residues and/or leakage， low adsorption capacities， low mass-transfer rates， and poor complex-substrate adaptabilities， which restrict their practical applications. Recent years have seen the introduction of metal/covalent-organic framework（MOF/COF） materials with high specific surface areas and adjustable pore sizes that have provided new avenues for improving MIP performance in a manner that significantly enhances adsorption capacity， the mass-transfer rate， and applicability. Consequently， MOF/COF-MIP composite materials are attracting increasing levels of attention because SPE adsorbents play important roles when screening and identifying NPs in coastal zones. Herein， we review recent advances in MOF/COF-MIP composite materials for use in SPE-pretreatment applications prior to chromatography or chromatography-MS for the screening and identification of NPs in coastal zones. Firstly， MIP synthesis methods are briefly summarized， and key factors， such as adsorption capacity， the binding kinetics mass-transfer rate， selectivity， and anti-interference ability， are discussed in detail. Adsorption capacity depends on recognition-site density and accessibility， which can be optimized through controlled polymerization and MOF/COF-MIP hybridization. The mass-transfer rate， which is influenced by surface hydrophilicity/hydrophobicity and pore architecture， dictates the analyte-diffusion rate to the binding sites. Advanced imprinting strategies， such as those involving restricted access materials（RAMs） and multi-template imprinting， are critical for minimizing nonspecific interactions in complex matrices. Imprinting and selectivity factors（IFs and SFs， respectively）， as key indicators， need to be rationally provided to deliver MIPs that are highly and reliably selective. In addition， this paper discusses methods used to prepare MOF-MIP and COF-MIP composites， focusing on the structural advantages of MOFs and COFs， including designability. MOF/COF-MIP syntheses leverage structural and functional synergies between the framework and the MIP. MOFs， with their metallo-organic coordination networks， serve as stable substrates for surface imprinting， whereas COFs， which contain covalent linkages， enable functional groups to be precisely tailored. Techniques such as radical polymerization and sol-gel processes are employed to balance crosslinking density and structural integrity. Room-temperature and microemulsion polymerization strategies enhance sensitive-template compatibility， thereby preserving recognition-site fidelity. Emerging methodologies include magnetic composite fabrication， streamlined separation processes， and improved operational efficiencies in field applications. MOF/COF-MIP-SPE coupled with CMS， which is typically used to detect coastal NPs， including POPs， antibiotics， EDCs， and microplastics， is summarized. These composites are highly proficient in isolating trace-level NPs from heterogeneous matrices by leveraging their tailored porosities and selective binding abilities. Enhanced chemical stability ensures consistent performance under fluctuating pH and high ionic-strength conditions， whereas the hierarchical pore structure facilitates rapid analyte transport. These molecularly imprinted SPE-based（MISPE-based） composites deliver detection sensitivities that surpass those of conventional SPE coupled with chromatography or chromatography-MS， which underscores their significant potential for efficiently enriching and purifying NPs in complicated coastal media. In addition， we discuss possible challenges faced when analyzing NPs in coastal samples using MOF/COF-MIP composite-based SPE， including regeneration-efficiency limitations， long-term stability under harsh conditions， and synthesis-protocol scalability. Repeated use in dynamic coastal environments often leads to pore fouling or MIP-layer degradation， necessitating durable composite design innovation. Looking forward， we discuss future prospects for the synthesis and application of composites， emphasizing the keen pursuit of multifunctional MOF/COF-MIP composites that are capable of simultaneously adsorbing， recognizing， enriching， and catalytically degrading NPs. Interdisciplinary approaches， especially ones that use artificial intelligence to energize and sustainably develop， including machine-learning-guided material design， while abiding by green-chemistry principles， have been proposed to accelerate the development of next-generation composites. Computational models capable of predicting framework-pollutant interactions can streamline the creation of new composites， while sustainable synthesis routes， such as those that employ aqueous solvents and biodegradable components， are expected to minimize environmental impacts. In conclusion， this review aims to bridge the gap between fundamental research and the practical applications of MOF/COF-MIPs for detecting NPs. By addressing current limitations through multidisciplinary collaboration and sustainable engineering， these materials are expected to be highly efficient， economical， environmentally friendly， portable， and industrializable； combined with SPE followed by chromatography or chromatography-MS， they hold significant potential for the screening and identification of NPs in coastal zones.

海岸带是海陆相互作用的交互地带，包括从陆地向海洋延伸的广阔区域，自海岸线向海陆两个方向辐射，包含沿海平原、沿海湿地、河口三角洲、潮间带、水下岸坡和浅海大陆区域，有效覆盖了地球表面的大约20%^［1］^。海岸带水体包括河口、海湾、近岸海域等多种类型，具有盐度变化大、生态系统复杂等特点；三角洲地区由于河流携带的大量泥沙和营养物质在此沉积，形成了复杂的生态环境，不仅溶解有各种盐类，还包含了众多来自陆地和海洋自身产生的有机和无机物质^［2，3］^；沉积物和土壤作为海岸带生态系统的重要组成部分，吸附着形形色色的物质，包括重金属、有机污染物以及微生物等，记录了长时间的环境变迁信息^［4，5］^；生物样品方面，水产品资源丰富如各种鱼类、贝类、虾类等，在海岸带生态链中处于关键位置，同时也可能成为污染物的富集载体^［6，7］^。沿海地区不仅是物质和能源交换最活跃和最敏感的区域，也是受人类活动影响大的区域，自然元素和生态过程的复杂性使得海岸带区域成为不同于一般陆地或海洋生态系统的独特系统^［8，9］^。随着人口快速增长和城市化进程加剧，大量污染物通过各种途径进入海岸带，沿海地区面临巨大环境压力^［10］^。功能退化、生态环境灾害频发，使得海岸带区域成为世界上污染最严重的地区之一，其特点是污染物类型多样、生态环境脆弱、恢复时间长^［11］^。近年来，新污染物^［12］^，如持久性有机污染物（persistent organic pollutants，POPs）^［13，14］^、抗生素^［15-17］^、内分泌干扰物（endocrine-disrupting chemicals，EDCs）^［18-21］^、微塑料及增塑剂^［22，23］^等受到越来越多的关注。这些新污染物具有独特的性质和多样的种类，在环境中的浓度往往较低，但生物累积性较强，部分还具有持久性和潜在的毒性，能够在环境中长时间存在并通过食物链不断传递和放大，其生态效应和环境健康影响远超传统污染物。目前，国内外对新污染物，尤其是海岸带、近海海域中的新污染物日益关注，研究持续升温^［12，24］^。

筛查与识别新污染物不仅是解析其环境归趋、评估健康风险及制定管控策略的基础，也对检测技术的灵敏度、选择性和分析通量等都提出了要求。在各种检测技术中，色谱、质谱及色谱-质谱联用技术展现出显著优势。主要包括，高效液相色谱-紫外检测法（HPLC-UV）、高效液相色谱-光电二极管阵列检测法（HPLC-PDA）、高效液相色谱-荧光检测法（HPLC-FLD）、高效液相色谱-质谱法（HPLC-MS）、超高效液相色谱-串联质谱法（UPLC-MS/MS）、液相色谱-高分辨质谱法（LC-HRMS）、气相色谱-火焰离子化检测器法（GC-FID）、气相色谱-质谱法（GC-MS）、气相色谱-串联质谱法（GC-MS/MS）、热解气相色谱-质谱法（Pyr-GC-MS）、热萃取解吸-气相色谱-质谱法（TED-GC-MS）等^［24］^。与此同时，由于海岸带新污染物大部分残留在沉积物、水和生物体中，基质复杂、含量低，因此还需要有效的样品前处理步骤，发展与之相匹配的样品前处理技术，以提高检测的灵敏度、准确性和可靠性，并保护仪器，确保其稳定运行。固相萃取（SPE）作为一种常用的样品前处理技术，能够有效富集目标物并去除干扰。在SPE过程中，吸附材料的选择直接影响萃取效果，其中分子印迹聚合物（molecularly imprinted polymers，MIPs）材料广受欢迎，分子印迹固相萃取（molecularly imprinted solid-phase extraction，MISPE）已广泛用于新污染物的测定中。MIPs作为一类人工合成的具有特异性识别能力的材料，效仿了酶-底物或抗原-抗体特异性结合的“锁-钥”机制，能够根据目标分子的形状、大小和功能基团进行定制化合成。在聚合过程中，引入模板分子，使其与功能单体通过非共价键或共价键结合，形成复合物。随后加入交联剂将复合物固定于聚合物网络中，形成与模板分子形状、尺寸及官能团互补的印迹空腔。洗脱模板分子后，聚合物中留下了特异性识别位点，可实现对目标分子或其结构类似物的高选择性识别和吸附^［25，26］^。

然而，MIPs材料仍存在吸附容量和传质效率等方面的局限，难以满足海岸带复杂基质中低浓度污染物的高效富集需求。针对这一挑战，通过将MIPs与金属有机框架（metal-organic frameworks，MOFs）和共价有机框架（covalent-organic frameworks，COFs）相结合，构建以MOFs/COFs为支撑骨架的MIPs复合材料，有望突破单一材料的性能瓶颈，综合各自优势获得具有优异性能的MOF/COF-MIPs复合材料。MOFs和COFs的多孔结构为MIPs提供了高比表面积的支撑，增加了吸附位点的数量，MIPs的特异性识别能力赋予了复合材料对目标污染物的高选择性，使其在复杂的海岸带环境中能够更有效地识别和富集新污染物，为海岸带新污染物的分析检测提供了新的有力工具和技术手段^［27，28］^。例如，Zhao等^［29］^通过在UiO-66-NH_2_表面包覆MIPs制备了新型MOF-MIPs复合材料，对氟喹诺酮类（fluoroquinolones，FQs）抗生素表现出高吸附容量、快传质速率和优异选择性，最大吸附容量为99.19 mg/g且在65 s内达到吸附平衡。Zou等^［30］^开发了具有方向选择性和三维空间选择性的新型COFs基复合材料（SiO_2_@F-COF@MIPs）。该材料对四溴双酚A（TBr-BPA）和四氯双酚A（TCl-BPA）具有良好的选择性吸附效果，最大吸附容量分别高至653.2 mg/g和158.8 mg/g。

本文综述了MOF/COF-MIPs复合材料用于海岸带新污染物筛查识别的研究新进展，主要内容如图1所示。首先概述了MIPs的制备技术和策略，着重讨论了MISPE中应考虑的吸附容量、传质速率、选择性和抗干扰能力等关键参数，接着归纳了改进MISPE性能的MOF/COF-MIPs制备方法及其提升作用。同时，总结了MOF/COF-MIPs复合材料结合SPE在测定海岸带水体、沉积物、土壤、生物样品、大气中POPs、EDCs、抗生素和微塑料等新污染物中的典型应用，展望了可能的挑战和发展前景。

**图1:**
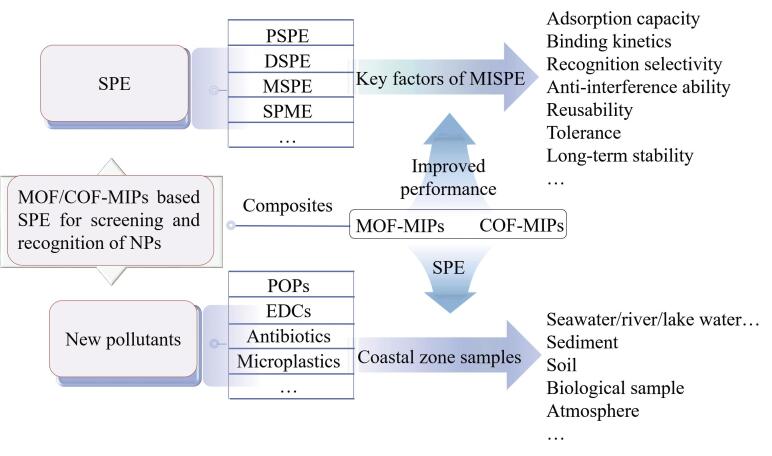
本文主要内容示意图

## 1 MISPE中需要考虑的关键因素

MIPs的制备过程涉及将模板分子与功能单体和交联剂通过特定的方法进行聚合，去除模板后形成具有特定识别位点的MIPs材料以捕获识别模板分子（目标物），满足分析检测中选择性的需求^［31］^。然而，MIPs在作为萃取相用于样品制备时，仍存在某些问题，包括结合动力学缓慢、吸附容量有限、模板渗漏以及同时识别多种靶标的能力低等。为克服这些局限，研究者们采用了表面印迹、纳米印迹技术及虚拟模板印迹、多模板印迹和多功能单体印迹策略等，或组合使用多种印迹技术和策略，结合自由基聚合和溶胶-凝胶聚合，以获得具有理想性能的MIPs^［32］^。MISPE从复杂样品中选择性识别和吸附目标物，进行富集和纯化，脱附和洗脱后进行分析测定^［33，34］^，主要包括装柱SPE（PSPE，通常称为SPE）、分散SPE（DSPE）、磁性SPE（MSPE）、固相微萃取（SPME）等多种模式^［32］^。优化提高MIPs的吸附容量、动力学传质速率、选择性、抗干扰能力等，结合SPE不同模式，以期显著提升MISPE的性能。

### 1.1 吸附容量

吸附容量是衡量MIPs性能的核心指标，指单位质量MIPs可吸附目标分析物的最大量，其高低直接影响实际应用中材料的使用效率和经济性。吸附容量与印迹腔的数量及其可及性有关，印迹腔的密度和空间分布直接影响结合位点的可利用性^［35］^。若印迹腔被非特异性位点覆盖或模板去除不完全（如残留模板占据空腔），将显著降低有效吸附容量。另外，功能单体与交联剂比例也会影响吸附容量，功能单体浓度过高可能导致过度交联，阻碍分析物扩散；浓度过低则无法形成足够稳定的结合位点。例如，Zhang等^［36］^通过优化丙烯酰胺（功能单体）与乙二醇二甲基丙烯酸酯（交联剂）的比例，使甲砜霉素MIPs的吸附容量显著提高到154.40 mg/g。高交联度虽增强了选择性，但容易降低印迹腔的可及性。因此，吸附容量与选择性之间的平衡是实际应用中的关键挑战。例如，Liu等^［37］^制备新型MIPs，既保留了高吸附容量（120.70 mg/g），又实现了对目标物的高选择性（IF=3.72）。

### 1.2 结合动力学传质速率

结合动力学传质速率反映了分析物从溶液扩散至MIPs结合位点的速度，直接影响实际应用中样品的处理效率。传质速率主要与以下因素密切相关：（1）表面修饰与亲疏水性。利用表面印迹技术制备亲水性涂层（如聚乙二醇）可减少表面黏附阻力，促进分析物迁移；疏水性表面则可能因吸附水分子形成扩散屏障^［38，39］^。（2）动态吸附平衡。结合位点的动态解离与再结合能力影响整体传质速率。例如，交联度高的MIPs虽选择性优异，但过强的结合力可能导致解离速率降低，延长吸附平衡时间^［40］^。（3）材料的多孔结构。孔径大小和比表面积决定分析物的传质效率。部分MIPs因孔道结构受限，使得大分子扩散效率降低，导致吸附平衡时间较长。引入多孔材料为基底（如MOFs、COFs或介孔二氧化硅）或设计分级孔结构（微孔-介孔协同）可显著提高MIPs的结合动力学传质速率^［41］^。

### 1.3 选择性

选择性对确保MIPs有效捕获目标分析物进而提升MISPE的纯化富集效率至关重要。MIPs的选择性源自其结合位点，这些位点能与模板分子的形状、大小和官能团精确匹配。无论何种类型的MIPs（有机、无机、杂化或生物基），其选择性都取决于结合位点内的均匀性和印迹腔内官能团的排列^［42］^。在聚合过程中，功能单体与模板的分子相互作用通过模拟计算可优化合成构型，而溶剂配比、温度/时间等变量也显著影响选择性^［43，44］^。例如，Han等^［45］^利用分子模拟方法结合化学计量选择合适的模板和单体合成MIPs，用于选择性萃取磺酰脲类除草剂，显示出高亲和力、选择性和低基质效应。MIPs通常需要用大量洗脱溶剂清洗，以确保模板分子完全去除。然而，长期溶剂暴露或高温处理（煅烧）等去除模板的方法可能导致结合位点失活，降低材料的选择性。为评估MIPs的选择性，通常计算印迹因子（IF）和选择性因子（SF）。但在此过程中存在两个常见错误：一是非印迹聚合物（NIPs）若省略模板清洗步骤，会因溶胀效应导致吸附能力异常偏低，造成IF虚高。二是在评估MIPs和NIPs对干扰化合物的吸附能力时，常使用未针对干扰化合物优化的色谱/质谱方法，导致测定的干扰化合物浓度偏低，可能误判为MIPs对这些化合物吸附能力低，使得SF虚高^［46］^。目前这两个常见问题并未引起足够重视，今后应加强实施，确保高选择性MIPs的IF和SF合理可靠。例如，制备NIPs时，除不添加模板分子，其余步骤与制备MIPs应保持完全一致，同样进行模板清洗步骤；在考察MIPs和NIPs对干扰化合物的吸附能力时，要使用针对特定干扰化合物优化后的色谱及色谱-质谱联用方法。

### 1.4 抗干扰能力

在实际样品分析中，目标分析物常与高浓度基质物质共存。这些物质可能通过占据MIPs印迹空腔或与目标物结合，干扰其识别、阻碍目标物向识别位点的迁移，甚至堵塞萃取柱/分离柱。同时，高盐度或极端pH值可能破坏MIPs结构，降低其在复杂样品中的利用率和稳定性。因此，优异的MISPE是色谱及色谱-质谱联用技术分析前的关键步骤。然而，由于MIPs骨架中官能团与干扰物间的非特异性相互作用难以完全消除，需通过选取合适的印迹策略制备MIPs以提升其抗干扰能力。鉴于MIPs的非特异性识别难以避免，采用有效印迹策略增强抗干扰能力至关重要^［47］^。例如，通过将限进材料（RAM）与MIPs结合，可解决此问题。RAM依据尺寸排除原则，允许小分子模板进入MIPs空腔而同时阻止大分子进入^［48，49］^，从而降低MIPs的非特异性识别，提高选择性。此外，设计包层策略^［50，51］^，即在MIPs的非印迹区涂覆功能惰性薄层（如TEOS）可增强抗干扰能力。

除上述4个需要加强关注的因素，MIPs在实际应用中仍面临一些问题。如：（1）模板分子残留与识别位点不均一，导致识别能力降低；洗脱不彻底或聚合物溶胀还可能导致残留模板污染样品。（2）耐储存性能有待考察。长时间的储存可能导致MIPs结构塌陷或官能团氧化。（3）重复使用性仍有待提高。多次使用后，MIPs的吸附容量和选择性可能因识别位点堵塞或结构塌陷而显著下降。

## 2 MOFs和COFs功能材料在MISPE性能提高中的应用

如前所述，将MIPs与MOFs和COFs材料相结合，不仅能在很大程度上突破传统MIPs的局限，还能引入高孔隙率、快速传质、抗干扰和高稳定性等特性，从而获得优异性能的MIPs复合材料，提高MISPE的实际应用效果。例如，MOFs和COFs的可调孔径在高效的分子识别中发挥重要作用；它们的孔径可以通过改变有机分子的长度来控制，在合成的过程中，可以利用不同性质的金属节点和有机分子进行化学裁剪^［28，52，53］^。

### 2.1 MOF-MIPs复合材料

MOFs是一类由无机金属中心与桥接有机配体相互连接而形成的具有周期性网状结构的晶体多孔材料，由金属离子或金属簇与有机配体通过自组装形成。它们具有可调控的孔径和丰富的功能基团，能够提供多种吸附位点和作用方式^［54］^。由于其高孔隙率、大比表面积和特殊的稳定性，MOFs可以作为MIPs的优良支撑材料^［55］^。MOFs结构中的金属离子或簇通过配位键形成三维框架，框架内高度有序的孔隙系统提供了大量的空间，使得MOFs能够在其表面和孔内承载更多的目标分子，从而提升MIPs对目标分子的加载能力，提高吸附容量。同时，MOFs中的金属节点可作为催化活性中心，赋予复合材料催化降解污染物的能力。

合成MOFs的方法包括溶剂热法、水热法、超声法等。在MOFs的合成过程中，多种变量都对材料的特性有所影响，如反应温度、溶剂组成和反应时间等参数会影响MOFs的结晶度、空隙规律性和表面活性。通过调节反应条件，能够制备具有不同结构和性能的MOFs以满足特定的应用需求^［56］^。通过在MOFs表面包覆MIPs，可以利用MOFs的多孔结构和MIPs的特异性识别能力，提高复合材料对目标污染物的吸附和分离性能。MOFs的刚性骨架可以有效防止MIPs变形和团聚，提高复合材料的稳定性和使用寿命^［57］^。同时，MIPs层对MOFs的高附着力，使其能够在MOFs表面形成具有高化学稳定性、机械稳定性和优异识别能力的印迹腔，因此能够很容易克服MIPs低比表面积、孔隙率和传质速率等缺陷，大大提高复合材料的效率，从而扩大其实际应用范围。

MOFs材料通常具有较高的化学和热稳定性，这对于在苛刻环境下使用MIPs非常重要，MOFs的引入可以帮助MIPs在不同的操作条件下保持高效性能。MOFs的化学稳定性表现在其对酸碱环境的耐受能力，有些MOFs材料具有高度耐酸碱的特性，特别是在极端酸性或碱性条件下，能够保持较好的稳定性，这意味着将MOFs与MIPs结合后，MIPs也能够在较为苛刻的环境中持续稳定。此外，MOFs材料的孔隙结构和框架的化学性质决定了它们在多种溶剂中（包括水、极性溶剂或有机溶剂）都能保持较高的稳定性。一些MOFs在极性溶剂中具有较高的稳定性，表明MOF-MIPs可以作为稳定的基底，不易被溶剂破坏，从而提高MIPs在各类溶剂环境中的使用寿命和可靠性^［55］^。

在制备MOF-MIPs复合材料时，需兼顾框架稳定性与印迹位点可及性。常用的MOFs有拉瓦希尔骨架（MIL）系列、奥斯陆大学（UiO）系列、类沸石咪唑酯骨架（ZIF）系列等，通常采用表面印迹技术和纳米印迹技术制备核壳结构^［58-60］^，聚合方式采用自由基聚合^［61］^和溶胶-凝胶聚合^［62］^。为提高MOFs的性能，常对其进行修饰，如引入功能性基团、改变金属离子、有机配体或核壳结构等，修饰后的MOFs可以提高对目标污染物的吸附能力和选择性，同时还可以增强其稳定性和生物相容性。尽管MOF-MIPs在吸附容量和催化性能上表现优异，其实际应用仍面临化学稳定性不足和传质受限的问题，部分MOFs（如ZIF-8）在高盐或酸性环境中易分解，需通过金属节点替换或表面钝化来提升耐受性。

### 2.2 COF-MIPs复合材料

COFs是一类由有机分子通过共价键连接形成的多孔框架材料，具有高度有序的孔道结构、良好的化学稳定性和可设计性^［63］^。COFs因其具有比表面积大、热稳定性高、孔径较窄以及密度较低等特性，被视作高吸附性能材料。与MOFs相比，COFs缺乏金属位点，功能主要依赖有机官能团修饰，但也避免了重金属浸出风险，更适合生物样品分析；COFs还具有更高的化学稳定性和可回收性^［64］^。COFs的合成方法包括离子热法、溶剂热法、微波辅助合成法等，不同的合成方法可以得到具有不同结构和性能的COFs。通过改变反应条件和选择不同有机配体，可制得具有不同孔径和功能基团的COFs，使其具有更大的应用潜力。为提高COFs的性能，常通过功能化修饰（如引入磁性纳米粒子）赋予其磁响应特性，从而实现对目标物的磁萃取分离^［52］^。

COFs材料的合成简单、孔径可控和可定制等优点，使其成为与MIPs集成的良好材料^［65］^。COFs的化学稳定性和可设计性使其在复杂的环境中具有良好稳定性，能够保证COF-MIPs复合材料的性能和使用寿命。通过设计和合成具有特定结构和功能的COF-MIPs复合材料，可以实现对目标污染物的有效吸附以及高选择性的识别、分离和富集。同时，如何将COFs的多孔限域效应与MIPs的特异性识别精准结合，仍是制备高性能COF-MIPs的一大挑战。可以通过优化聚合策略，开发适配COF-MIPs的合成方法，例如，目前常用的微乳液聚合^［66］^和室温合成^［67］^等。室温合成避免了制备过程中使用高温煅烧的方法和复杂的印迹步骤，降低了能耗，使得COFs的合成过程具有良好的可控性，但功能单体的高纯度需求仍制约其大规模应用。未来需开发低成本生物基单体和连续流生产技术等以推动实际应用。

## 3 MOF/COF-MIPs-SPE结合色谱/质谱在海岸带新污染物筛查识别中的应用

MIPs与功能材料的集成不仅能够解决传统MIPs的关键局限性，而且还可以引入独特的功能以增强MIPs复合材料的性能和实际样品适用性。MOFs和COFs多孔结构的设计、可微调和功能化使它们有望被广泛应用^［68-70］^。与MIPs类似，MOFs和COFs的几何结构也具有孔隙和空腔，因此，利用MOF/COFs作为多孔基质来合成MIPs，可以通过增加孔数、比表面积和识别能力来显著提高复合材料的性能。MOF/COF-MIPs材料结合SPE技术进行海岸带样品前处理，采用色谱或色谱-质谱技术进行测定，能够在海岸带POPs、EDCs、抗生素、微塑料和增塑剂等各类新污染物的筛查识别和检测中发挥重要作用。

### 3.1 MOF-MIPs-SPE用于海岸带新污染物筛查识别

基于新污染物种类和结构的多样性，研究者合理设计和制备了相应的MOF-MIPs，用作SPE吸附剂，结合色谱/质谱，进行了海岸带介质中新污染物的筛查识别和检测研究。典型示例如下。

Rahimpoor等^［60］^以MIL-101（Fe）为载体、二嗪农为模板分子，采用一锅法表面纳米印迹技术制备了MIL-101（Fe）@MIP，该纳米复合材料通过针捕装置（NTD）对空气中的二嗪农进行了采样、提取，结合GC-FID测定，检出限（LOD）和定量限（LOQ）分别为0.02 μg/m^3^和0.1 μg/m^3^。同时，该方法的重复性和重现性分别在3.9%~5.1%和5.1%~6.4%范围内，证明其具有高精度。研究显示该方法与推荐的美国国家职业安全卫生研究所（NIOSH）方法之间存在较高的相关系数（*R* ^2^=0.978 1）。利用该方法对实际条件下农业工作环境大气中的二嗪农进行了采样和测定，结果表明，基于MOF@MIP的NTD结合GC-FID方法能够作为一种快速、简单、环保的方法，用于大气中二嗪农类POPs的取样和测定，也有望为海岸带POPs检测及其相关的健康和环境监测提供新思路。

Weng等^［58］^以恩诺沙星（ENR）为模板分子、甲基丙烯酸为功能单体、乙二醇二甲基丙烯酸酯为交联剂，采用表面印迹技术和沉淀聚合制备了ZIF-67负载表面印迹聚合物（ZIF-67@MIPs），制备过程如图2所示。考察了对ENR的吸附性能，结果表明在30 min内达到吸附平衡，最大吸附量为9.02 μg/mg，印迹因子为2.58。该MOF-MIPs用作SPE填料（图2），结合UPLC-MS/MS检测，获得了低的LOD（0.23 ng/mL）和低的LOQ（0.76 ng/mL），方法灵敏度高。湖水中的加标回收率为83.79%~100.68%、相对标准偏差（RSD）为4.46%~7.35%，表明方法准确可靠。因此，该MOF-MIPs-SPE为海岸带水体中痕量ENR的精准测定提供了行之有效的前处理材料和技术。

**图2:**
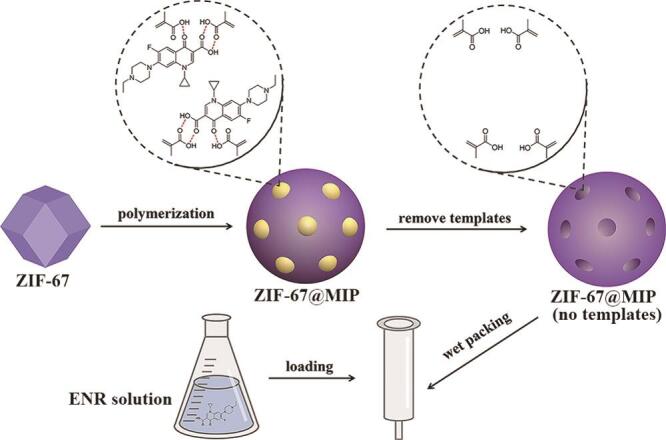
ZIF-67@MIPs及SPE柱制备流程图^［58］^

Li等^［59］^采用表面印迹技术合成了新型核壳结构沸石咪唑酸酯框架-8@分子印迹聚合物（ZIF-8@MIPs），对有机磷农药（OPPs）具有特异性识别和快速吸附能力。结合HPLC-MS/MS测定，在1~200 µg/L范围内具有良好线性（*R* ^2^≥0.992 7）。在农产品（花椰菜、萝卜、梨、甜瓜）中添加3种不同浓度水平时，回收率为82.5%~123.0%，RSD小于8.24%。在最佳条件下，建立的基于ZIF-8@MIPs复合材料的SPE-HPLC-MS/MS方法灵敏、方便、高效，可用于农产品中OPPs的测定。该研究不仅提供了一种检测OPPs的新方法，也为设计具有极快传质速率的新型吸附剂进行其他新污染物筛查识别提供了一种很有前途的策略。

Li等^［71］^采用可逆加成-断裂链转移聚合技术在MOFs表面成功制备了一种新型限进介质印迹纳米材料（RAM-MIPs），将其用于DSPE，结合HPLC-UV分析了未经其他预处理的牛奶与河水中的FQs。所得材料具有良好的结合量（60.81 mg/g）、快速的结合动力学（15 min）、令人满意的选择性以及良好的消除基质干扰的能力。在最优化条件下，利用该方法对牛奶和典农河河水中的多种FQs残留进行检测，获得了令人满意的线性关系（*R* ^2^>0.998 8）、较低的LOD（牛奶中为1.02~3.15 μg/L，河水中为0.93~2.87 μg/L），回收率分别为80.7%~103.5%和85.1%~105.9%、RSD分别不高于5.3%和4.7%。该研究显著提升了复杂基质中痕量FQs的富集效率与检测灵敏度，有望为海岸带介质中其他新污染物的精准测定提供兼具实用性与稳定性的新方法。

Zhang等^［72］^提出了一种简单通用的逐层制备MOF-MIPs复合材料的策略，制备了磁性金属有机骨架（MMOFs）负载的MIPs纳米粒子（MMOFs@MIPs），用于MSPE，结合HPLC-UV测定实际样品中的双酚A（BPA），材料制备、MSPE和测定过程如图3a所示。该材料的形貌表征如图3b所示，与Fe_3_O_4_相比，Fe_3_O_4_@ZIF-8具有更粗糙的表面和更大的颗粒尺寸，在Fe_3_O_4_表面上可以看到许多紧密间隔的立方ZIF-8晶体，为负载MIPs层提供了大的比表面积。结合了Fe_3_O_4_、MOFs和MIPs的优点，该MOF-MIPs展示出优异的磁性能、快速的传质速率、良好的选择性吸附能力。该MMOFs@MIPs-MSPE-HPLC-UV对BPA质量浓度的线性范围宽至4个数量级（0.5~5 000 μg/L），LOD低至0.1 μg/L；将材料用于未受污染的柠檬汁、罐头山楂和矿泉水样品的分析，得到了88.3%~92.3%的回收率。这项工作提供了一种快速、高富集能力的新材料和技术，在海岸带水体和食品样品新污染物分析中富有应用潜力。

**图3:**
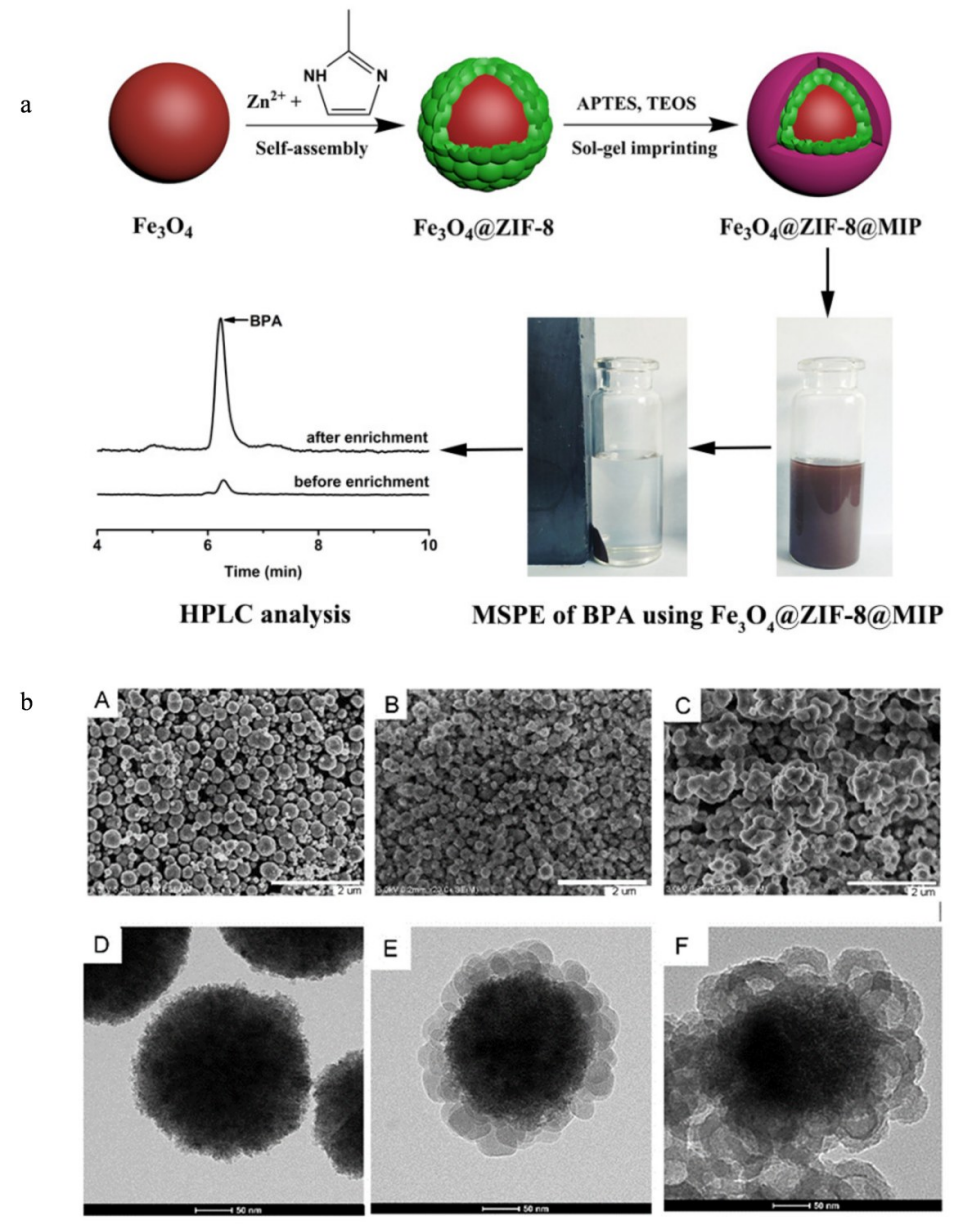
（a）Fe_3_O_4_@ZIF-8@MIP萃取富集双酚A及结合HPLC测定的应用示意图；（b）材料的SEM和TEM图像^［72］^ A： SEM image of Fe_3_O_4_； B： SEM image of Fe_3_O_4_@ZIF-8； C： SEM image of Fe_3_O_4_@ZIF-8@MIP； D： TEM image of Fe_3_O_4_； E： TEM image of Fe_3_O_4_@ZIF-8； F： TEM image of Fe_3_O_4_@ZIF-8@MIP.

Li等^［73］^合成了Fe_3_O_4_@ZIF-8@MIPs，用于对黄河水样中四溴双酚A（TBr-BPA）的选择性吸附，并用HPLC进行分析。该吸附剂结合了MMOFs提供的大比表面积和丰富的孔隙以及MIPs的特异性识别位点，由于材料具有磁芯，因此可以方便快速地分离和重复利用。在最佳条件下，该复合材料在15 min内达到117.6 mg/g的最大吸附量，回收率为88.28%~107.07%、RSD小于2%，结果满意。该研究为海岸带水体中新污染物的高效富集和去除提供了一种性能优异的新材料和MSPE技术。

Mirzajani等^［74］^以孕酮分子为模板制备MIPs，将MIPs、氧化石墨烯和ZIF-8在一根薄纤维上，制备了一种MIPs复合材料多单片纤维（MMF），用于SPME，通过HPLC对孕酮、睾酮、*β*-谷甾醇、胆固醇和油菜甾醇进行分离测定。该方法成功应用于鱼肉、鸡肉、蛋黄和蔬菜样品中5种分析物的测定，在0.01~1 000 μg/L范围内线性关系良好，LOD为3~5 ng/L，相对平均回收率为95.0%~101.0%。该MMF-SPME可直接用于不同真实样品中多种EDCs的测定，为海岸带复杂基质中新污染物测定提供了新思路。

目前关于MOF/COF-MIPs提取海岸带微塑料的研究缺乏，但对于增塑剂的研究成为一大热点。一般来说，样品中邻苯二甲酸酯（PAEs）的检测需要经过预处理，通常采用常规的液-液萃取（LLE）和SPE方法，然后采用GC或HPLC检测PAEs。LLE存在有机溶剂用量高、选择性差、环境污染严重等一系列缺点。而常规SPE耗时较长，步骤多样，选择性较差^［75］^。此外，这些化合物在食品/饮料中的浓度非常低。因此，发展高选择性、快速、高效的样品前处理技术对食品和环境中微量PAEs的分离和富集非常必要^［75，76］^。例如，Gao等^［77］^以邻苯二甲酸二丁酯（DBP）为模板分子，采用一锅法表面印迹，基于ZIF-7，制备了一种新型MIPs复合材料（MIP@ZIF-7），结合SPE-HPLC法对食品样品中4种PAEs进行提取和测定。结果表明，MIP@ZIF-7结合了ZIF的高比表面积和优异的稳定性以及识别的特异性，对PAEs具有更高的吸附容量和选择性。在最佳条件下，该方法的线性范围为1~100 μg/L，LOD为0.1~0.8 μg/L，饮用水、碳酸饮料和果汁饮料中PAEs的加标回收率为77.1%~100.7%，RSD为1.65%~4.87%（*n*=3）。该基于MIP@ZIF-7的SPE结合HPLC-UV法在检测海岸带超痕量PAEs的实际应用中具有良好的潜力。

Zhang等^［23］^以DBP和邻苯二甲酸二甲酯（DMP）为双模板，合成了一种新型杂化材料（ZIF-7-NH_2_@MIP），将其填充在SPE柱中，结合HPLC测定4种PAEs。该方法在加标10 μg/L的矿泉水、碳酸饮料和果肉果汁3种实际样品中的回收率为88.68%~98.58%、RSD为1.19%~3.40%，表明方法灵敏、准确。该杂化材料结合了氨基官能化ZIF-7-NH_2_优异的水热性和大的比表面积以及双模板MIPs同时识别多种目标物的能力，有望为海岸带水体中痕量PAEs测定提供高效的样品前处理手段。

Li等^［78］^以DBP为模板制备了一种新型磁性MIPs复合材料（Fe_3_O_4_@MOF@MIP-160），具有Fe_3_O_4_@MOF载体，通过将MSPE与先进的功能新材料巧妙结合，开发出一种新型MSPE技术，用于食品中痕量PAEs的高效、快速、选择性提取，并通过GC-MS进行检测。该材料对DBP和邻苯二甲酸二乙己酯（DEHP）具有良好的识别和吸附能力，吸附量分别为260 mg/g和240.2 mg/g，吸附速率快（约20 min达到平衡）。此外，Fe_3_O_4_@MOF@MIP-160可以回收6次，与传统的SPE材料相比，成本低，易于操作，节省时间。对饮用水、果汁和白葡萄酒中的PAEs含量进行了分析，回收率为70.3%~100.7%。该研究建立的方法适用于食品基质中PAEs的检测和去除，并为发展适用于海岸带新污染物的更高效、简单和环境友好的检测方法提供了可能。

### 3.2 COF-MIPs-SPE用于海岸带新污染物筛查识别

针对不同结构和种类的新污染物，可分别制备相应的COF-MIPs作为SPE吸附剂，结合色谱/质谱，实现海岸带介质中新污染物的筛查识别与测定，如图4所示。

**图4:**
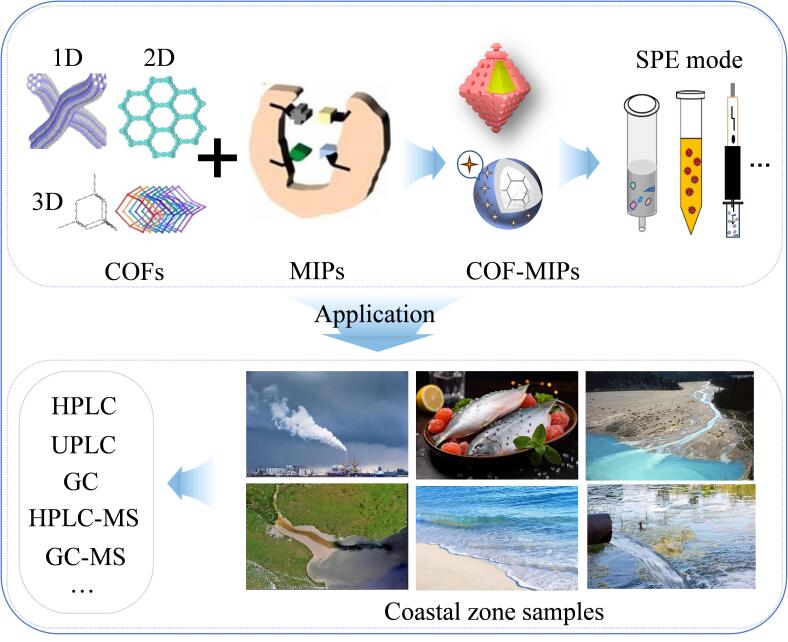
COF-MIPs的制备、SPE模式及结合色谱/质谱在海岸带样品检测中的应用

Zou等^［30］^以TBr-BPA为模板，首次合成了一种具有方向选择性和三维空间选择性的新型COF基MIPs复合材料（SiO_2_@F-COF@MIPs），使用HPLC-UV进行分析。复合材料作为吸附剂对TBr-BPA和类似化合物TCl-BPA具有良好的选择性吸附效果，最大吸附容量分别为653.2 mg/g和158.8 mg/g。通过计算机模拟和实验分析，探讨了SiO_2_@F-COF@MIPs高选择性吸附的机理，结果表明，TBr-BPA在SiO_2_@F-COF@MIPs上的吸附主要基于卤素键相互作用、三维空间选择性效应以及氢键和静电相互作用。将SiO_2_@F-COF@MIPs作为SPE吸附剂用于湖水、沱江水、自来水等实际水样和鱼类样品中TBr-BPA和TCl-BPA的富集，获得了满意的回收率。该方法在0.50~100 μg/mL范围内对3种水样中的TBr-BPA和TCl-BPA均呈现良好线性关系（*R* ^2^范围为0.999 0~0.999 2），LOD和LOQ分别为0.20和0.50 μg/mL，回收率范围为74.26%~102.33%。在鱼样中，两种化合物在质量浓度为0.50~50 μg/mL时可获得良好的线性关系（*R* ^2^范围为0.998 2~0.998 7），LOD和LOQ分别为0.30和0.50 μg/mL，回收率为77.35%~107.33%。此外，日内RSD和日间RSD分别在0.27%~2.39%和0.06%~6.64%范围内。这些结果表明，开发的SiO_2_@F-COF@MIPs材料适用于多种实际样品中TBr-BPA和TCl-BPA的同时提取，在高选择性富集海岸带新污染物方面应用前景广阔。

Xiang等^［79］^以双酚AF为虚拟模板、NiFe_2_O_4_为磁芯，利用对苯二甲醛（TPA）和1，3，5-三（4-氨基苯基）苯（TAPB）之间形成席夫碱以制备疏水分子印迹磁共价有机框架（MI-MCOF）。这种有机框架显著减少了传统印迹聚合的时间消耗，并避免了使用传统的引发剂和交联剂，合成的MI-MCOF在水和尿液样品中具有良好的磁响应性和稳定性，并对BPA具有较高的选择性和动力学特性。BPA在MI-MCOF上的平衡吸附容量为50.65 mg/g，比其3种结构类似物高3~7倍。BPA的印迹因子高达3.17，3种类似物的选择性系数均>2.0，表明所制备的纳米MIPs复合材料对BPA具有优异选择性。基于MI-MCOF纳米复合物的MSPE与HPLC-FLD相结合，获得了宽线性范围（0.1~100 μg/L）和低LOD（0.020 μg/L），在河水、自来水、饮料和人体尿液样品中的回收率高（83.5%~110%）、RSD为0.5%~5.7%。结果表明，MI-MCOF-MSPE-HPLC-FLD方法在取代传统的磁分离和吸附材料的同时，为复杂基体如海岸带介质中高效提取BPA提供了新思路。

Su等^［80］^以ENR为模板分子制备了一种新型吸附剂MI-COFs，通过多巴胺简易印迹法在不可逆COFs表面产生印迹腔，以选择性提取鱼肉、虾肉等海岸带生物样品中的FQs。该COF-316中的氰基基团被水解为羧基，作为结合位点，与多巴胺的氨基相互作用并吸附FQs。此外，利用多巴胺自聚合在COF-316表面产生聚多巴胺（PDA）印迹层，用于快速和选择性识别FQs。在MI-COFs的基础上，发展了DSPE结合HPLC-FLD的方法，同时测定ENR、诺氟沙星（NOR）和环丙沙星（CIP），该方法获得了低检出限（0.003~0.05 ng/mL）和高精密度（RSD小于3.5%）。MI-COFs对ENR的最大吸附容量为581 mg/g，与NI-COFs相比，吸附能力提高了2.2倍。在鱼肉样品中，检出NOR和ENR分别为6.4 μg/kg和13.9 μg/kg，而在虾、鸡和猪肉样品中未检出该3种FQs，加标回收率为80.4%~110.7%。因此，该MI-COFs-DSPE法在海岸带食品样品分析中具有良好应用潜力。

Tang等^［81］^设计和制备了一种新的含C-C键的3D COF，命名为JNU-7，以甲基丙烯酸为功能单体、L-精氨酸为虚拟模板、乙二醇二甲基丙烯酸酯为交联剂，制备得到基于JNU-7的MIPs（JNU-7-MIP），进而建立了基于JNU-7-MIP的装柱SPE结合HPLC-MS的方法，以精确测定微囊藻毒素（MC-LR）。与JNU-7-NIP相比，JNU-7-MIP对虚拟模板L-精氨酸具有更高的吸附容量（156 mg/g）和更快的吸附动力学（20 min）。在优化条件下测定土壤样品和湖水样品中的MC-LR，检出限为0.008 ng/mL，线性范围为0.025~100 ng/mL，加标回收率为90.5%~106.5%。此外，JNU-7-MIP能在30 min内迅速将不同水样中的MC-LR从1 mg/L去除至0.26~0.35 μg/L，远低于世界卫生组织（WHO）规定的1 μg/L MC-LR的饮用水安全限值。该研究展示了基于3D COF的MIPs作为新型吸附剂在环境污染物测定和去除方面的巨大应用潜力，为海岸带新污染物监测和治理提供了新的思路与工具。

Su等^［82］^采用一锅反应合成分子印迹COFs，该COF-MIPs能够调整框架的形状和构象以适应模板分子，对黄曲霉毒素（AFTs）具有高选择性识别能力。此外，具有丰富印迹位点和功能基团的COF-MIPs的吸附量为258.4 mg/g，是COFs-NIPs的3倍。基于该COF-MIPs的SPE-HPLC对AFTs的检出限低至0.003~0.09 ng/mL，精密度好，RSD≤6.7%，在稻谷、玉米、小麦和花生样品中的加标回收率为85.4%~105.4%。该COF-MIPs的高选择性使得水产品中AFTs的准确测定成为可能，为适于不同新污染物的COF-MIPs的定制化合成提供了一条简便可行的新途径。

MOFs/COFs-MIPs复合材料已展现出效率高、选择性好和可循环利用等优点，在各类实际水样、沉积物和水产品中进行的应用研究表明，这些复合材料能够有效地从复杂基质中分离和富集目标物，为后续的色谱或色谱-质谱分析提供可靠的前处理，确保测定结果的灵敏、准确。

## 4 结论与展望

MOF/COF-MIPs复合材料在海岸带水体、土壤/沉积物、生物样品、大气等各种复杂介质的样品前处理中取得了显著效果，结合色谱或色谱-质谱测定，在海岸带新污染物筛查识别和检测中显示出巨大的应用潜力。当前，这两类复合材料的制备和应用，尤其是针对海岸带新污染物的研究仍相对较少。今后应加强材料制备，从MOFs/COFs和MIPs各自着手及协同考虑，进行制备方法探索和合成条件优化，关注材料的吸附容量、选择性、再生能力和成本控制等，进而拓展MISPE在海岸带介质中的应用范围。然而，相关方法的实际应用效能仍受限于材料结构或功能适配性不足、复杂环境响应机制不明确等关键瓶颈，需通过跨维度协同优化实现突破。

（1）新污染物种类多样、性质各异，针对不同污染物的化学结构与作用机制，需要选择不同的功能单体、印迹方法及MOFs/COFs框架等获得定制化的高性能MIPs复合材料。虽然理论上，各种结构的MOFs/COFs都可用以制备MOF/COF-MIPs复合材料，但实际研究和应用发现，不同的结构可能更适合于某些特定的场景。

MOF/COF-MIPs材料各有优劣势。例如，MOFs的介孔设计更灵活，COFs以微孔为主，对疏水小分子吸附效率较高。金属盐和有机配体成本较低，而高纯度有机单体价格昂贵，从而COFs原料成本普遍高于MOFs。MOFs的金属位点可增强氢键或离子交换作用，COFs的共价官能团可以定向修饰、精准识别，故MOFs对金属离子吸附通常更好，而COFs对极性分子选择性更强。部分MOFs降解可能释放金属离子，而COFs降解产物为有机小分子，环境风险较低，终端处理更为环保。总之，MOF-MIPs和COF-MIPs各具特色，MOFs具多功能性与良好的传质效率，COFs长于化学稳定性和精准设计；实际应用中需根据目标污染物性质（如分子尺寸、极性）、基质复杂性（如盐度、pH）及海岸带复杂情况（如潮汐压力、生物扰动）合理选材。在需多功能集成、大分子污染物处理、动态环境响应或磁性分离的情况下，可考虑优先选用MOF-MIPs材料；在需高化学稳定性、精准小分子识别、生物相容性或极端环境的应用中，可考虑COF-MIPs材料^［68，83］^。今后的研究应该加强关注哪种结构适合哪个领域或者不同的结构对应不同的性能特征，确保复合材料的结构满足特定性能需求。

（i）对于POPs，采用含卤素或疏水基团的功能单体（如乙烯基吡啶），通过*π-π*作用增强对芳香族POPs的识别；引入羧基或氨基单体，通过氢键和静电作用提升对抗生素极性分子的吸附能力；设计含酚羟基或双键的单体（如甲基丙烯酸），模拟目标物的空间构型来提高选择性以筛选EDCs；对于微塑料与增塑剂，可利用疏水性单体（如苯乙烯）结合MOFs的孔隙限域效应，增强对疏水污染物的富集效率。相对于庞杂的目标物，目前可用的功能单体仍然十分有限，如何快速选择及制备合适的功能单体是MOF/COF-MIPs制备所面临的重要挑战，需要继续加强计算方法及相关数据库的使用和开发。

（ii）提高MOF/COF-MIPs材料的吸附容量、传质速率、选择性及抗干扰能力，是提高MISPE性能的重要考量参数。组合使用多种印迹技术和策略及探索新的印迹技术策略和自由基聚合、溶胶-凝胶聚合等，例如通过虚拟模板、多模板或多功能单体策略制备广谱性材料以同时捕获多种新污染物，选用不同MOFs如MIL、UiO、ZIF系列并进行修饰，以提高MOFs吸附能力、稳定性和生物相容性；同时，可制备不同COFs如二维、三维、层状、异构并进行修饰，以及采用微乳液聚合、室温合成等方法，以期提高性能。

（2）发展先进的合成技术和开展绿色化制备。MOF/COF-MIPs复合材料的合成需多步反应（如MOFs/COFs框架构建、模板分子印迹及洗脱、功能单体聚合等），今后加强开发一步法原位合成技术。例如，通过原位自组装策略^［84］^或界面限域聚合^［85］^等方法，将MOFs/COFs的生长与MIPs的印迹步骤集成，以降低多步反应带来的风险和成本。此外，探索使用生物基前驱体如纤维素^［86］^等，替代传统的合成配体，并深度发展水相/室温合成路线^［87］^，将有望推动MOF/COF-MIPs复合材料的绿色规模化生产。同时，注重发展绿色印迹^［88］^，例如开发低能耗、无模板残留的印迹技术，减少有毒试剂使用；通过温和洗脱，实现材料循环再生，降低处理成本。此外，结合催化降解技术（如光催化MOFs），将吸附的新污染物转化为低毒产物，推动“吸附-检测-降解”一体化应用，实现污染物的资源化利用。采用实验设计、响应曲面法等手段系统优化MOF/COF-MIPs合成参数及MISPE条件，也遵循了绿色化学原则。

（3）海岸带样品极为复杂，基质中含有大量的无机离子、有机物、微生物等，且温度、盐度、pH值等环境条件变化较大，样本常处于动态过程。这些复杂因素可能会干扰MOF/COF-MIPs复合材料与目标物的相互作用，影响其吸附容量和选择性等。因此，要重点考虑提高复合材料在海岸带样品中的适用性、抗干扰能力和再生循环能力。如Liu等^［89］^采用皮克林乳液模板法首次合成了一种新型MOF-MIPs微球（EIM），通过减少基质干扰和提高复杂基质中的纯化效率（93.93%），有效提高了EIM的特异性识别能力和吸附容量。Nizamidin等^［90］^使用紫外光照射方法在铌酸锂（LiNbO_3_）薄膜复合光波导（COWG）衬底表面生长，将螺恶嗪嵌入Nb-MOF框架中大大提高了选择性，获得了具有雪花印迹表面的SP@Nb-MOF膜COWG新型高效MOF-MIPs材料。

部分极端高盐度环境可能导致盐离子与目标污染物竞争结合位点，降低MIPs复合材料的吸附效率。可以考虑在材料表面引入疏水涂层，或设计对目标污染物具有强亲和力的螯合基团，并加强对耐盐框架的开发探索，避免结构腐蚀导致框架坍塌。通过惰性骨架设计和抗菌功能化，抵抗酸碱侵蚀、减少生物污染，提升材料的长期稳定性与抗老化能力。结合光催化自清洁、电化学再生和磁性分离等技术，还有望实现材料的高效再生与循环利用。

（4）传统色谱或色谱-质谱仪器设备体积大，实验室依赖性强，需要推动检测设备的小型化和智能化发展，以满足现场、快速、准确检测的需求。如采用便携GC-MS仪结合网络遥感能够实现现场检测，增强方法通用性。将MOF/COF-MIPs填充至微型固相萃取柱进行现场富集，结合便携GC-MS仪，有望实现对海岸带样品的现场乃至原位分析。应重视MISPE小柱和分子印迹膜片的规模生产和商业化应用，以推动MOF/COF-MIPs功能材料更好地服务于新污染物的筛查与识别。

（5）大数据和人工智能技术的快速发展为MOF/COF-MIPs在海岸带新污染物检测中的应用提供了重要助力。利用大数据技术，可以整合大量的检测数据，建立污染物的分布模型和环境行为预测模型，为环境监测和管理提供科学依据。利用机器学习可以预测功能单体与污染物结合能，加速材料筛选与优化进程。人工智能算法，如机器学习、深度学习等^［91，92］^，能够对检测数据进行分析和处理，提高数据的解析能力，实现对海岸带复杂基质中多种新污染物的同时精准识别和灵敏检测，有望促进后续的环境行为及减控等的研究。

（6）MIPs的特异识别机理和精准作用机制目前仍然不够明确，需要加强探索。更深入地理解MIPs的识别机理和阐明作用机制，就能够更好地可控制备，获得满足需求的MOF/COF-MIPs复合材料。开发易制备、低成本、高稳定性、高吸附容量、适应复杂海岸带介质的MOF/COF-MIPs材料势在必行。

此外，通过理解MOFs与COFs机械性能的化学本质及环境影响，可综合MOFs的金属活性与COFs的共价稳定性，形成MOF-COF复合材料^［93］^，有望实现性能互补。随后引入MIPs，得到的MOF-COF-MIPs复合材料应具有稳定性高、选择性好、吸附能力强、传质快等优点。然而，目前鲜有MOF-COF-MIPs复合材料相关研究的报道，今后可尝试制备该三合一复合材料用于海岸带新污染物的测定。

总之，需要融合各类技术不断探索优化MOF/COF-MIPs材料的设计和合成，提高在海岸带介质中的适用性，助力色谱或色谱-质谱灵敏、精准测定新污染物。同时，结合高效、经济、环保、便携及产业化等因素进行考虑，MOF/COF-MIPs复合材料样品前处理结合色谱/质谱技术在海岸带新污染物筛查识别中必将发挥日益重要的作用。

## 作者团队简介

海岸带生态环境分析监测与生态修复课题组，隶属于中国科学院烟台海岸带研究所。自2009年建组以来，课题组聚焦海岸带生态环境监测和治理，通过化学与地学等多学科交叉融合，创建“学科交叉-技术创新-应用示范”的全链条科技创新体系，在生态环境监测技术研发、污染防控技术突破、生态修复工程示范等领域形成特色优势，持续为海岸带可持续发展提供关键科技支撑。致力于面向海岸带复杂系统的生态环境安全问题，发展陆海统筹的海洋生态环境分析监测理论与在线监测技术，主要以典型生态环境要素（生态、环境和灾害要素）为研究对象，利用纳米材料、生物材料以及光电磁等分析探测技术，构建微纳分析传感界面，探索分析监测新原理、新方法和新仪器技术；关注典型污染物（纳米颗粒污染、新型污染物等）与生命体系（生物大分子、活性物种等）作用研究，揭示其与人类健康相关性；发展基于微生物在环境监测与生态修复中的新技术，服务于陆海统筹的海洋污染治理、生态修复与海岸带可持续发展。研究涉及化学（化学测量学、环境化学）和地学（海洋科学、海洋化学）等领域。

相关网站：http：//www.yic.ac.cn/yjsjy/yjsdsjj/201809/t20180911_5075602.html

**Figure:**
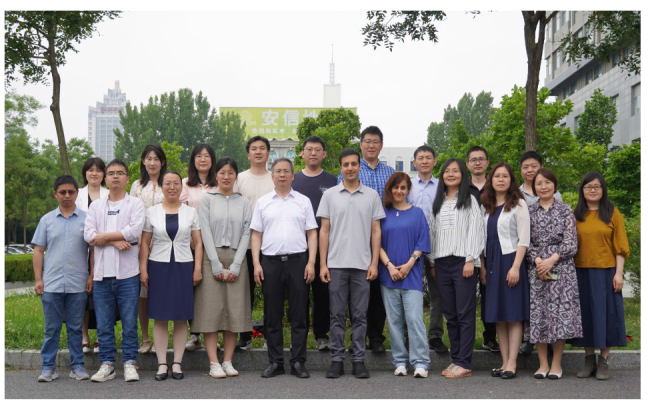


### 人才队伍

**课题组组长：**陈令新研究员

**职工及学生：**研究员5人，副研究员4人，高级工程师2人，助理研究员6人，工程师4人，博士后及研究生20余人

**团队精神：**创新海岸带科学观测新技术，支持海岸带可持续发展

### 科研项目及成果

**科研项目：**国家重点研发计划、国家自然科学基金重大科研仪器研制项目、国家自然科学基金、山东省重点研发计划（重大科技创新工程）项目、中国科学院海洋大科学研究中心重点部署项目、中国科学院知识创新工程、山东省科技型中小企业创新能力提升工程项目、山东省自然科学基金重点项目等

**科研成果：**出版《海洋环境分析监测技术》《海洋生态分析监测技术》《表面增强拉曼散射光谱技术》等中、英文著作5部，团队发表论文520余篇（其中SCI期刊论文480余篇）。获“一种海水营养盐在线监测系统及海水营养盐检测方法”“一种开放海域沉积物污染的修复方法”等授权中国发明专利70余项。团队工作对保护海岸带生态环境安全、构建海岸带科学体系做出开创性贡献

**获奖情况：**海洋工程科学技术奖、中国分析测试协会科学技术奖、山东省自然科学奖、烟台市科学技术奖等

### 研究领域

1. 海岸带复杂生态环境分析方法（基础研究、新原理与新方法）：创新化学测量技术进行污染物识别、分析监测与环境毒理评估。a） 样品前处理材料与方法（结合色谱、质谱或色谱-质谱测定）——核心技术：分子印迹聚合物、金属/共价有机框架等功能材料； b） 成像可视化分析方法（光学探针）——核心技术：表面增强拉曼光谱分析、分子探针、纳米光学探针

2. 海岸带复杂生态环境监测技术（仪器研制、系统集成与平台技术）：创新海洋测量技术进行生态环境监测、监视与灾害预警。a） 现场快速检测试剂盒、分析监测仪器微型化——核心技术：微流控芯片、纸基微流控芯片、侧流式试纸、流动注射分析； b） 集成多参数在线监测技术（长时续立体监测、观测网建设）——核心技术：基于湿法化学的流动注射分析进行多参数营养盐、总磷、总氮、重金属等的监测； c） 可视化监测监视技术——核心技术：水下视频、三维成像海洋光学成像进行浮游生物、有害菌、病毒等的成像分析

3. 陆海统筹海洋污染与治理技术（生态修复基础研究与工程技术）：近海环境污染过程、环境毒理、生态毒理与生态修复。a） 海洋新型污染环境行为、环境毒理效应与生态效应模拟研究； b） 基于生物/化学材料的海洋污染水体和沉积物的环境修复、以微生物为主体的联合生态修复技术

### 仪器设备

**实验室科学研究仪器：**全二维气相色谱-高分辨质谱仪、气相色谱-三重四极杆质谱仪、气相色谱仪、液相色谱-三重四极杆质谱仪、超高效液相色谱仪、液相色谱-离子阱质谱仪、多类型拉曼光谱仪等

**自主研发仪器设备：**海水营养盐原位在线监测系统、走航式海水营养盐在线监测系统、海洋浮游生物光学成像系统、总磷总氮在线监测系统、滨海湿地水环境多参数在线监测系统、海洋牧场多水层水质在线监测系统、海水温度链在线监测系统等
